# Supplementary material for: A hypothalamic circuit links hunger to mesolimbic dopamine to drive feeding
Source: bioRxiv. 2026 Oct 30:2026.09.10.750730. Originally published 2026 Sep 15. Preprint. [Version 2] doi: 10.64898/2026.09.10.750730 (PMC13596576; doi:10.64898/2026.09.10.750730)
Supplement: Supplementary file 1 [file NIHPP2026.09.10.750730v2-supplement-2.pdf]

bioRxiv preprint doi: https://doi.org/10.64898/2026.09.10.750730; this version posted October 30, 2026. The copyright holder for this preprint (which was not certified by peer review) is the author/funder, who has granted bioRxiv a license to display the preprint in perpetuity. It is made available under a CC-BY 4.0 International license.

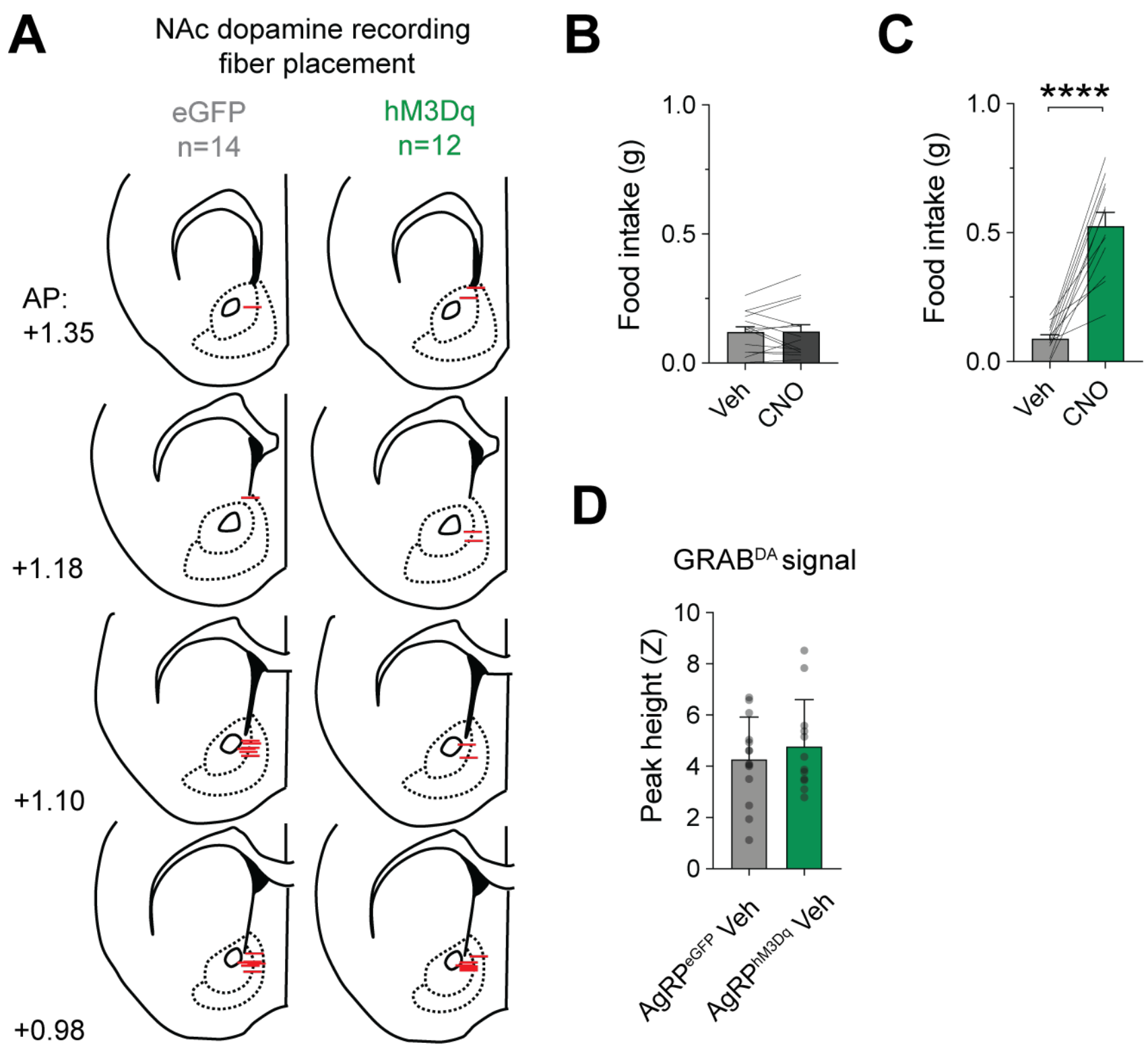

Figure S1. Fiber placements, functional controls, and comparable dopamine responses between AgRP^{eGFP} and AgRP^{hM3Dq} mice

(A) Fiber placements in the NAc in control (AgRP^{eGFP}) mice (n = 14) and experimental (AgRP^{hM3Dq}) mice (n = 12).

(B) Food intake following vehicle (Veh) or Clozapine-N-Oxide (CNO) administration in AgRP^{eGFP} mice (n = 14, paired *t*-test, $p = 1.0$).

(C) Food intake following Veh or CNO administration in AgRP^{hM3Dq} mice (n = 12, paired *t*-test, $p < 0.0001$).

(D) Peak NAc dopamine response in the two minutes following vehicle i.p injection in AgRP^{eGFP} and AgRP^{hM3Dq} mice (eGFP, n = 14; hM3Dq, n = 12; unpaired *t*-test, $p = 0.4612$).

Data are presented as mean ± SEM. *$p < 0.05$, **$p < 0.01$, ***$p < 0.001$, and ****$p < 0.0001$.

bioRxiv preprint doi: https://doi.org/10.64898/2026.09.10.750730; this version posted October 30, 2026. The copyright holder for this preprint (which was not certified by peer review) is the author/funder, who has granted bioRxiv a license to display the preprint in perpetuity. It is made available under a CC-BY 4.0 International license.

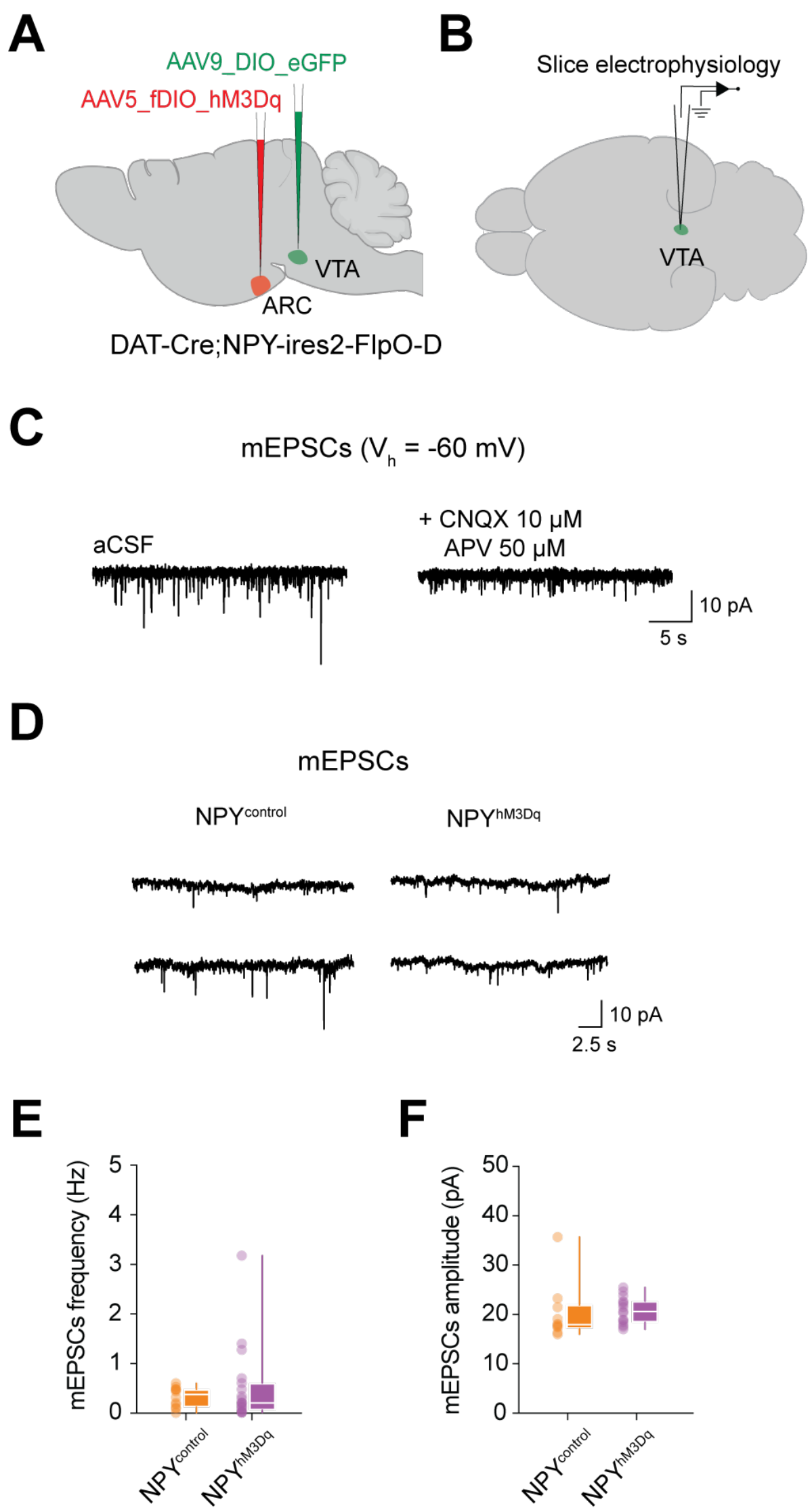

Figure S2. AgRP neuron stimulation does not change mEPSCs on VTA dopamine neurons

(A) Schematic depicting chemogenetic activation of NPY/AgRP neurons and labeling of VTA dopamine neurons with eGFP in DAT-Cre;Npy-ires2-FlpO-D mice.
(B) Schematic depicting ex vivo whole-cell electrophysiological recordings from VTA dopamine neurons following in vivo chemogenetic stimulation of NPY/AgRP neurons.
(C) Representative miniature excitatory postsynaptic currents (mEPSCs) recorded as inward currents are blocked by the glutamate receptor antagonists CNQX and APV.
(D) Representative mEPSC (two cells per group) from VTA dopamine neurons in $NPY^{control}$ and NPY^{hM3Dq} mice.
(E) mEPSC frequency in VTA dopamine neurons from $NPY^{control}$ (n = 12 cells from 6 mice) and NPY^{hM3Dq} (n = 19 cells from 7 mice) mice (Mann-Whitney test, U = 108, $p = 0.823$).
(F) mEPSC amplitude in VTA dopamine neurons from $NPY^{control}$ (n = 12 cells from 6 mice) and NPY^{hM3Dq} (n = 19 cells from 7 mice) mice (Mann-Whitney test, U = 99, $p = 0.192$).

Data are presented as mean ± SEM. *$p < 0.05$, **$p < 0.01$, ***$p < 0.001$, and ****$p < 0.0001$.

bioRxiv preprint doi: https://doi.org/10.64898/2026.09.10.750730; this version posted October 30, 2026. The copyright holder for this preprint (which was not certified by peer review) is the author/funder, who has granted bioRxiv a license to display the preprint in perpetuity. It is made available under a CC-BY 4.0 International license.

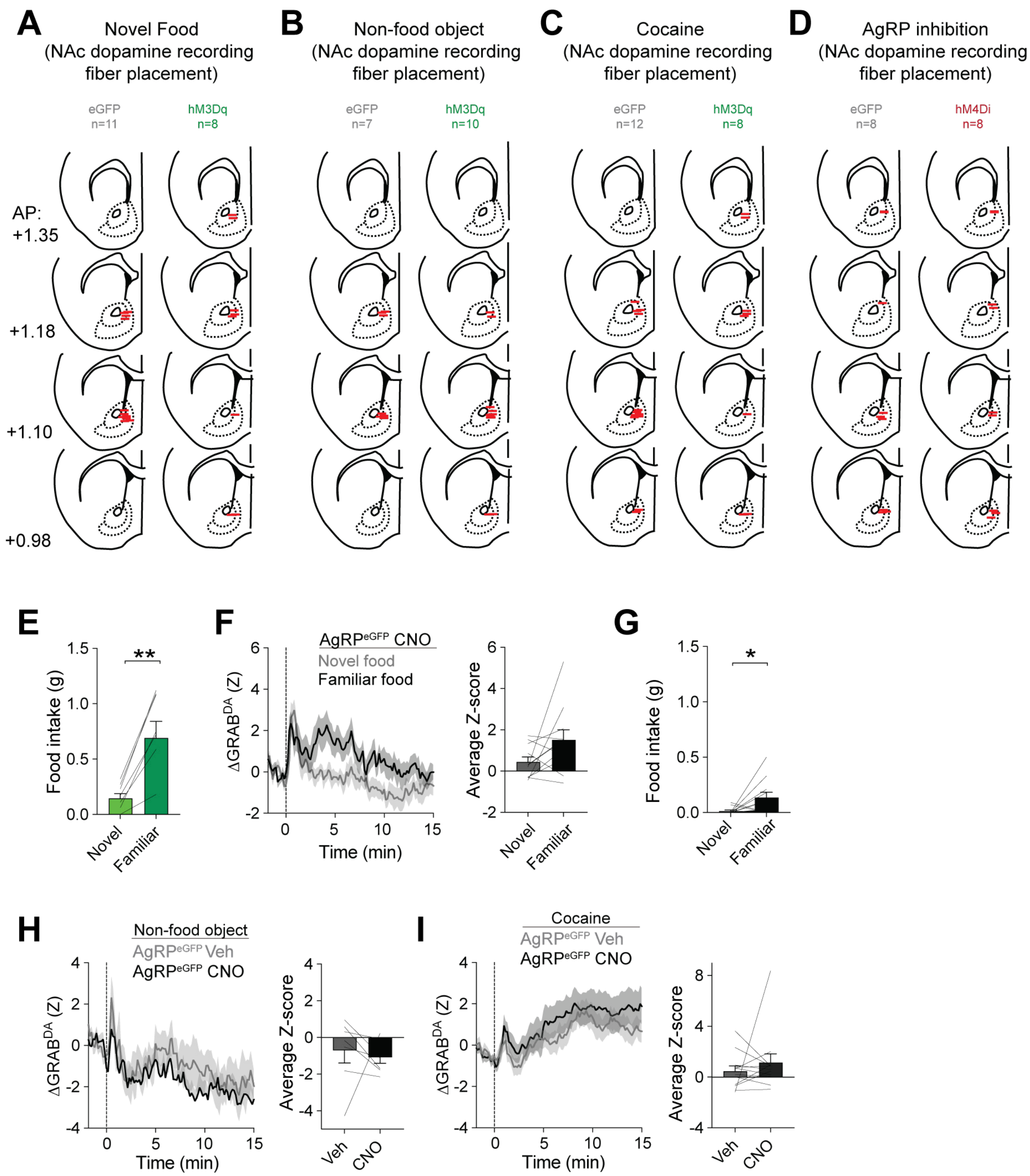

Figure S3. Fiber placements and control measurements for stimulus-evoked NAc dopamine responses

(A-D) Fiber photometry placements in the NAc for mice used for novel food **(A)**, non-food object **(B)**, cocaine **(C)**, and AgRP neuron inhibition **(D)** experiments.

(E) Food intake during exposure to novel food vs. familiar food in $AgRP^{hM3Dq}$ mice (n = 8, paired *t*-test, $p = 0.0017$).

(F) NAc dopamine signals (left) and average Z-score (right) during the first and second exposure to novel food in control $AgRP^{eGFP}$ mice (n = 11, paired *t*-test, average Z-score, $p = 0.0831$).

(G) Food intake during exposure to novel food vs. familiar food in $AgRP^{eGFP}$ mice (n = 11, paired

bioRxiv preprint doi: https://doi.org/10.64898/2026.09.10.750730; this version posted October 30, 2026. The copyright holder for this preprint (which was not certified by peer review) is the author/funder, who has granted bioRxiv a license to display the preprint in perpetuity. It is made available under a CC-BY 4.0 International license.

t-test, $p = 0.0463$).

(H) NAc dopamine signals (left) and average Z-score (right) to presentation of a non-food object following Veh or CNO administration in control AgRP^{eGFP} mice (n = 7, paired *t*-test, average Z-score, $p = 0.6814$).

(I) NAc dopamine signals (left) and average Z-score (right) to cocaine following Veh or CNO administration in control AgRP^{eGFP} mice (n = 12, paired *t*-test, average Z-score, $p = 0.3892$).

Data are presented as mean ± SEM. *$p < 0.05$, **$p < 0.01$, ***$p < 0.001$, and ****$p < 0.0001$.

bioRxiv preprint doi: https://doi.org/10.64898/2026.09.10.750730; this version posted October 30, 2026. The copyright holder for this preprint (which was not certified by peer review) is the author/funder, who has granted bioRxiv a license to display the preprint in perpetuity. It is made available under a CC-BY 4.0 International license.

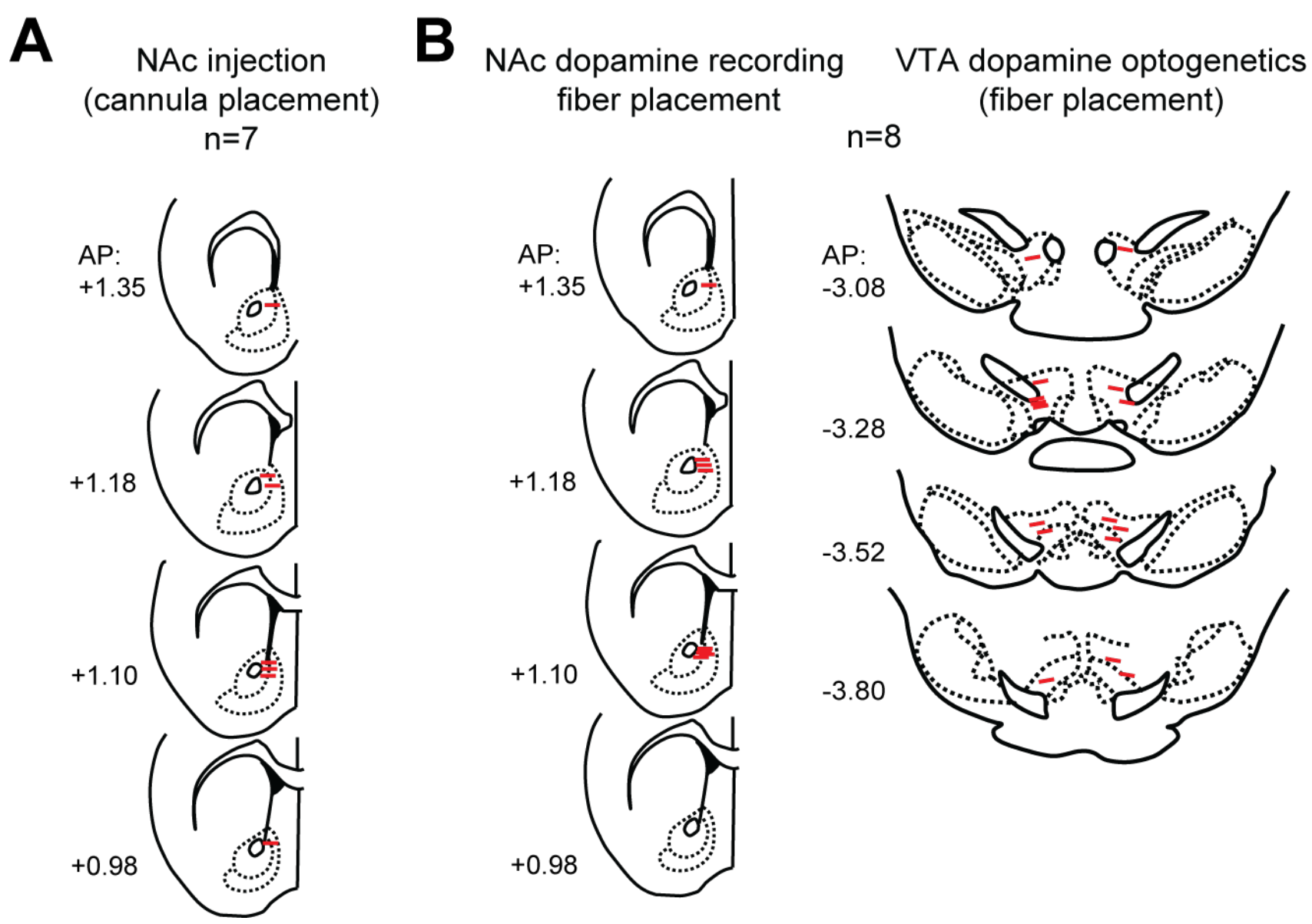

Figure S4. Cannula placements for dopamine antagonist experiments and fiber placements for dopamine inhibition experiments

(A) Cannula placements in the NAc for dopamine antagonist experiments.

(B) Fiber placements in the NAc and optogenetic fiber placements in the VTA for VTA dopamine inhibition experiments.

bioRxiv preprint doi: https://doi.org/10.64898/2026.09.10.750730; this version posted October 30, 2026. The copyright holder for this preprint (which was not certified by peer review) is the author/funder, who has granted bioRxiv a license to display the preprint in perpetuity. It is made available under a CC-BY 4.0 International license.

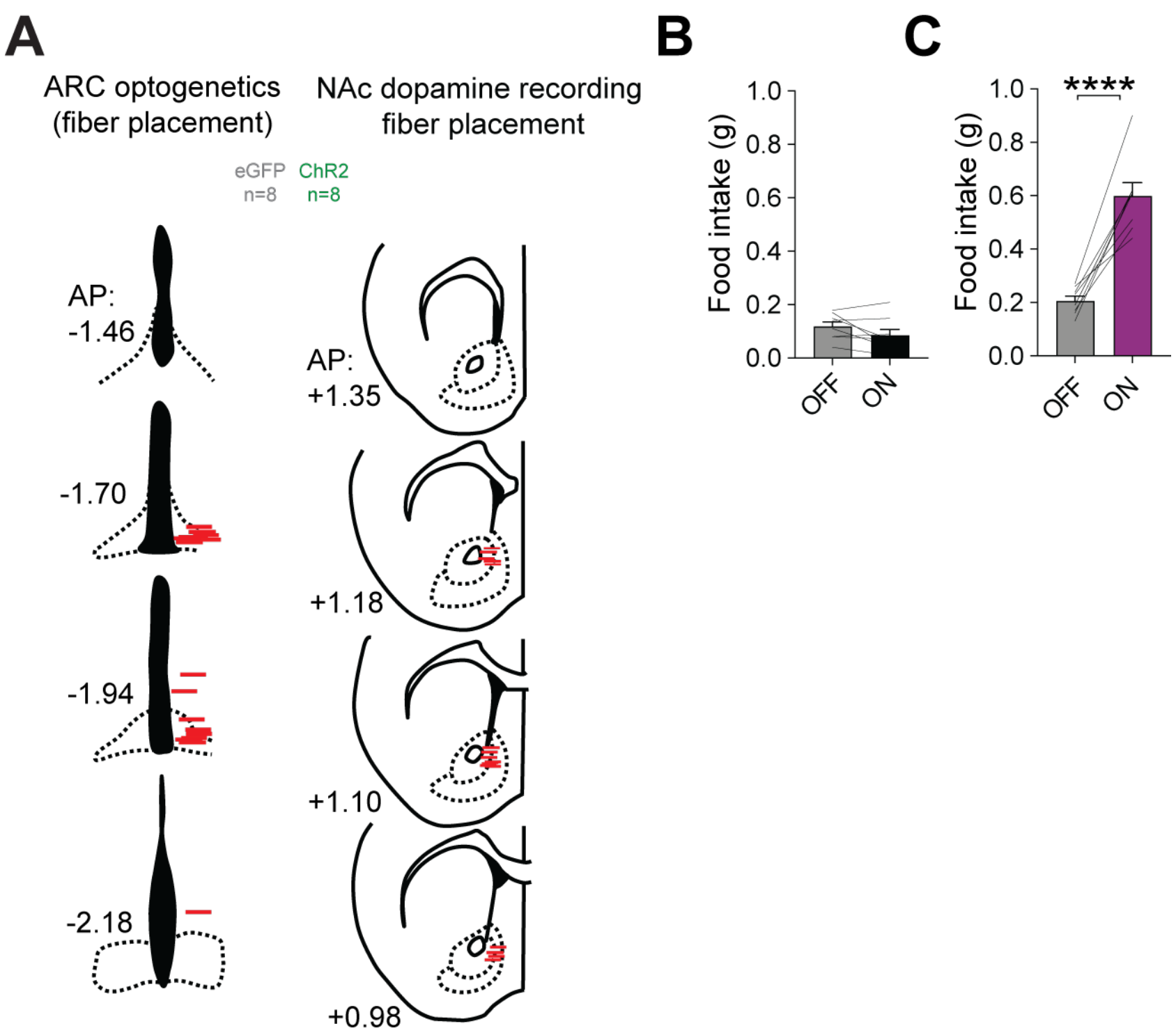

Figure S5. Fiber placements and positive control measurements for optogenetic AgRP neuron stimulation and dopamine recordings
(A) Fiber placements for optogenetic stimulation of AgRP neuron cell bodies in the arcuate nucleus (ARC) and GRAB-DA fiber photometry recordings in the nucleus accumbens (NAc).
(B) Food intake with optogenetic stimulation OFF or ON in control $AgRP^{eGFP}$ mice (n = 8, paired *t*-test, $p = 0.1263$).
(C) Food intake with optogenetic stimulation OFF or ON in $AgRP^{ChR2}$ mice (n = 8, paired *t*-test, $p < 0.0001$).

Data are presented as mean ± SEM. *$p < 0.05$, **$p < 0.01$, ***$p < 0.001$, and ****$p < 0.0001$.

bioRxiv preprint doi: https://doi.org/10.64898/2026.09.10.750730; this version posted October 30, 2026. The copyright holder for this preprint (which was not certified by peer review) is the author/funder, who has granted bioRxiv a license to display the preprint in perpetuity. It is made available under a CC-BY 4.0 International license.

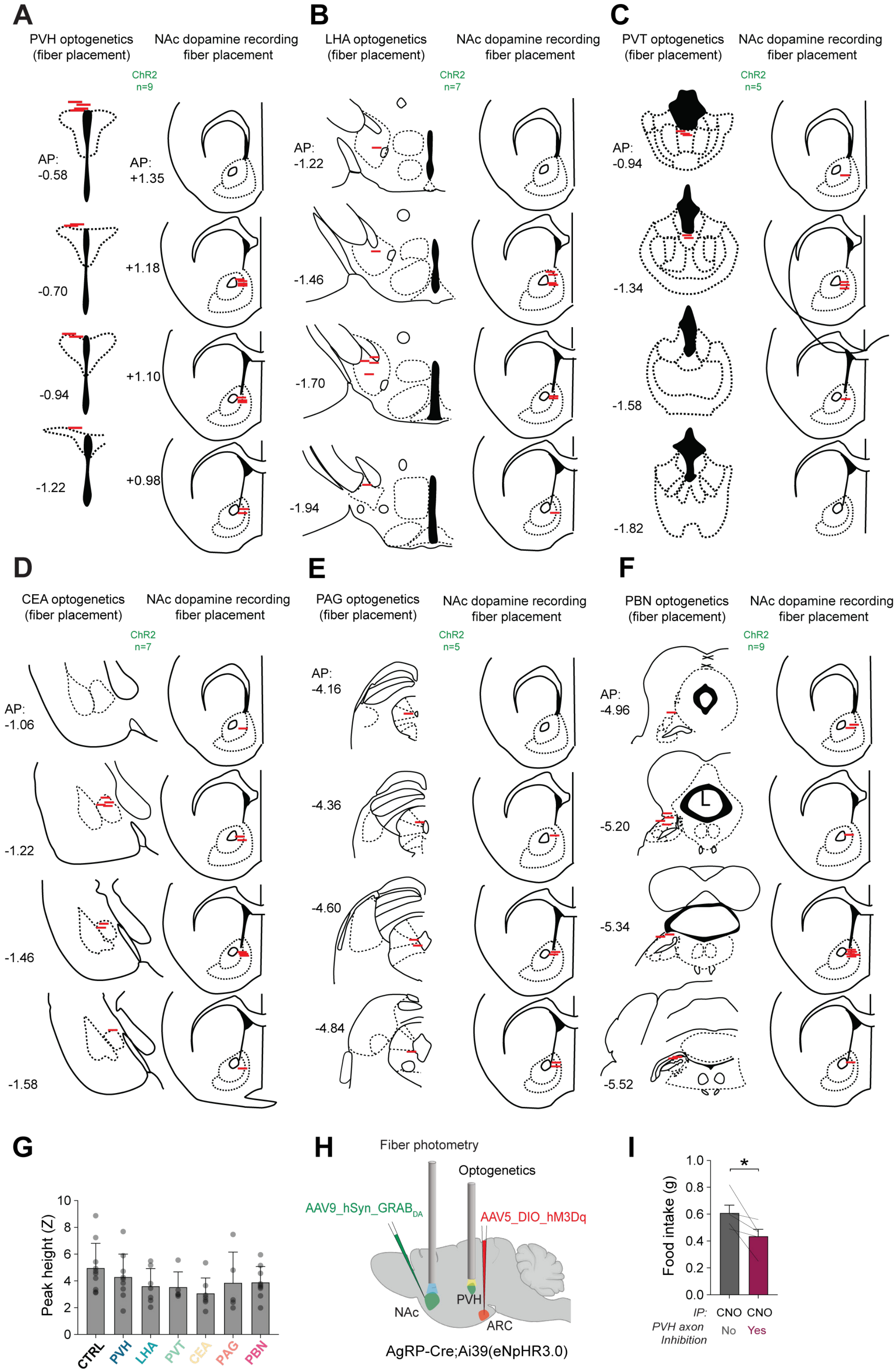

817

bioRxiv preprint doi: https://doi.org/10.64898/2026.09.10.750730; this version posted October 30, 2026. The copyright holder for this preprint (which was not certified by peer review) is the author/funder, who has granted bioRxiv a license to display the preprint in perpetuity. It is made available under a CC-BY 4.0 International license.

Figure S6. Fiber placements and control data for AgRP axon projection manipulations.
(A-F) Optogenetic and photometry fiber placements for experiments stimulating AgRP neuron axon projections to the paraventricular hypothalamus (PVH, **A**), lateral hypothalamic area, (LHA, **B**), paraventricular thalamus (PVT, **C**), central amygdala (CEA, **D**), periaqueductal gray (PAG, **E**), and parabrachial nucleus (PBN, **F**).
(G) Peak NAc dopamine response following vehicle i.p injection in controls and AgRP neuron axon stimulation mice (n = 52, one-way ANOVA, $F(6, 45) = 1.125$, $p = 0.3633$).
(H) Schematic depicting NAc GRAB-DA fiber photometry during chemogenetic activation of AgRP neurons and simultaneous optogenetic inhibition of AgRP axon terminals in the PVH.
(I) Food intake during AgRP neuron activation with or without inhibition of AgRP axon terminals in the PVH (n = 5, paired *t*-test, $p = 0.0446$)

Data are presented as mean ± SEM. *$p < 0.05$, **$p < 0.01$, ***$p < 0.001$, and ****$p < 0.0001$.

bioRxiv preprint doi: https://doi.org/10.64898/2026.09.10.750730; this version posted October 30, 2026. The copyright holder for this preprint (which was not certified by peer review) is the author/funder, who has granted bioRxiv a license to display the preprint in perpetuity. It is made available under a CC-BY 4.0 International license.

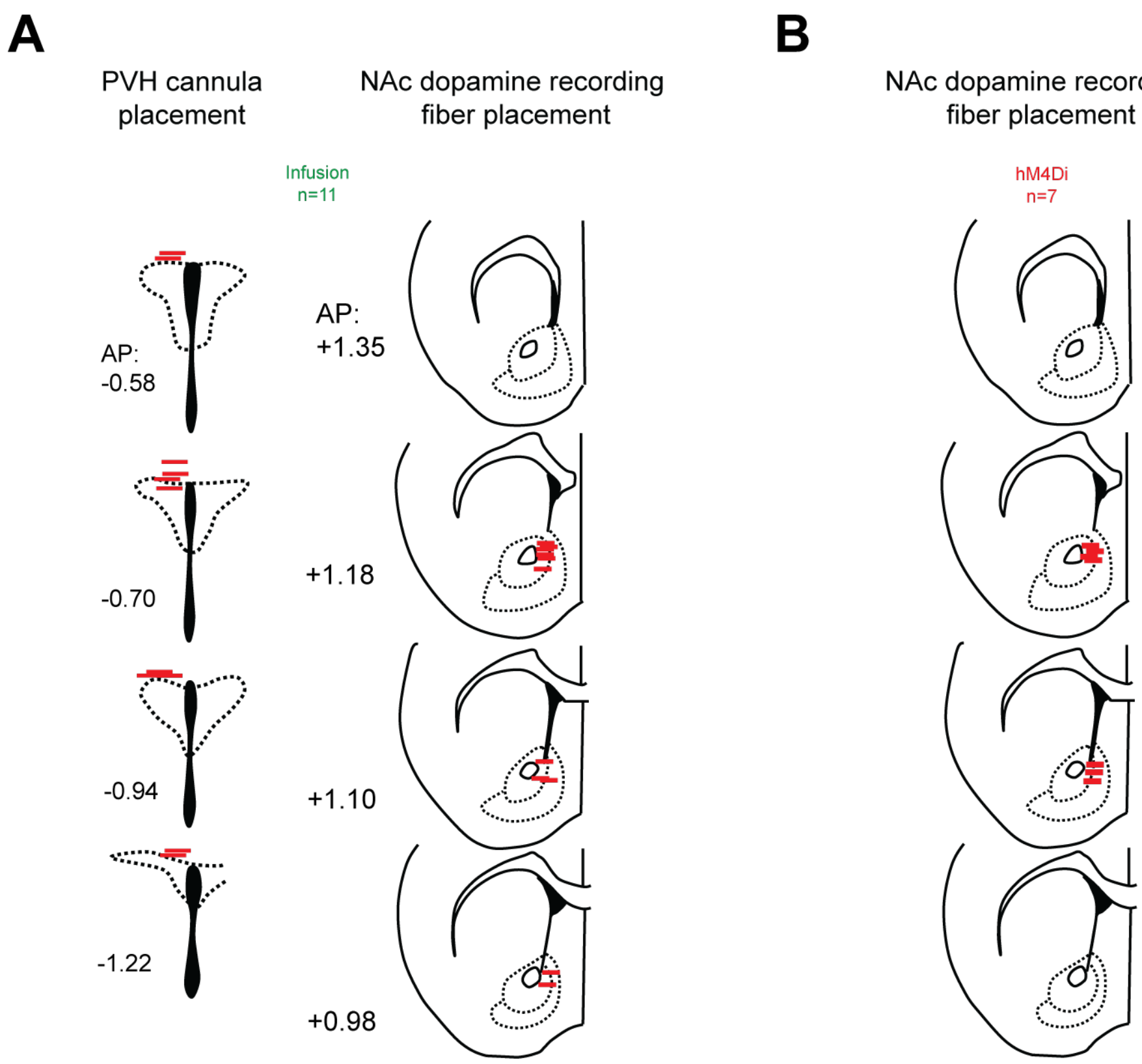

Figure S7. Cannula and dopamine fiber photometry placements for PVH manipulation experiments.
(A) PVH cannula placements and NAc photometry fiber placements for PVH pharmacology experiments.
(B) NAc photometry fiber placements for PVH Y1R neuron inhibition experiments.
